# Outcome of experimental porcine circovirus type 1 infections in mid-gestational porcine foetuses

**DOI:** 10.1186/1746-6148-7-64

**Published:** 2011-10-21

**Authors:** Dipongkor Saha, David J Lefebvre, Richard Ducatelle, Jan V Doorsselaere, Hans J Nauwynck

**Affiliations:** 1 Laboratory of Virology, Faculty of Veterinary Medicine, Ghent University, Belgium; 2 Department of Pathology, Bacteriology and Poultry Diseases, Faculty of Veterinary Medicine, Ghent University, Belgium; 3 Department of Health Care and Biotechnology, KATHO Catholic University College of South-West Flanders, Belgium; 4 Veterinary and Agrochemical Research Centre, Virology Department, Unit of Vesicular and Exotic Diseases, Brussels, Belgium

## Abstract

**Background:**

Porcine circovirus type 1 (PCV1) has been described as a non-cytopathic contaminant of the PK-15 cell line. Several experimental infections with PCV1 failed to reproduce disease in pigs. Therefore, PCV1 is generally accepted as non-pathogenic to pigs. To our knowledge, nothing is known about the outcome of PCV1 infections in porcine foetuses. This was examined in the present study.

**Results:**

Nine foetuses from three sows were inoculated at 55 days of gestation: three with 10^4.3 ^TCID_50 _of the PCV1 cell culture strain ATCC-CCL33, three with 10^4.3 ^TCID_50 _of the PCV1 field strain 3384 and three with cell culture medium (mock-inoculated). At 21 days post-inoculation, all 6 PCV1-inoculated and all 3 mock-inoculated foetuses had a normal external appearance. Microscopic lesions characterized by severe haemorrhages were observed in the lungs of two foetuses inoculated with CCL33. High PCV1 titres (up to 10^4.7 ^TCID_50_/g tissue) were found in the lungs of the CCL33-inoculated foetuses. All other organs of the CCL33-inoculated foetuses and all the organs of the 3384-inoculated foetuses were negative (< 10^1.7 ^TCID_50_/g tissue) by virus titration. PCV1-positive cells (up to 121 cells/10 mm^2 ^in CCL33-inoculated foetuses and up to 13 cells/10 mm^2 ^in 3384-inoculated foetuses) were found in the heart, lungs, spleen, liver, thymus and tonsils. PCR and DNA sequencing of *Rep *recovered CCL33 or 3384 sequences from CCL33- or 3384-inoculated foetuses, respectively.

**Conclusions:**

From this study, it can be concluded that cell culture PCV1 can replicate efficiently and produce pathology in the lungs of porcine foetuses inoculated at 55 days of foetal life.

## Background

Porcine circovirus type 1 (PCV1) is a small, non-enveloped circular single-stranded DNA virus of the family *Circoviridae*. PCV1 was originally detected as a non-cytopathic contaminant of the PK-15 cell line, ATCC-CCL33 [1]. PCV1 infections are widely distributed around the world as described before [2-4]. Seroprevalence of PCV1 at herd level varies between 10% [5] and 100% [6]. Although PCV1 DNA has been isolated from lymph nodes of a piglet in France with a wasting condition [7], it is generally accepted that PCV1 is non-pathogenic to pigs [8-13]. Experimental infections with PCV1 failed to reproduce disease in pigs [8,9,14]. The distribution of PCV1 in different pig tissues after experimental infections has been demonstrated [9]. PCV1 has been detected in cases of congenital tremors in newborn pigs and aborted/stillborn piglets, indicating the possible occurrence of vertical transmission of PCV1 [9,15-17]. In contrast, no evidence of PCV1 infection was found in piglets affected with congenital tremors in an 11 years retro-prospective study [18]. To our knowledge, nothing is known about the outcome of PCV1 infections in porcine foetuses.

In the present study, the virological and pathological outcomes were examined in porcine foetuses that were experimentally inoculated with PCV1 at 55 days of gestation.

## Methods

### Viruses

Two different PCV1 strains were used in this study. The PCV1 cell culture strain CCL33, was originally detected as a non-cytopathic contaminant of the PK-15 cell line [1,19]. The PCV1 field strain 3384 was isolated from stillborn piglets [9]. Both PCV1 strains have been sequenced and their full genomic sequences have been deposited in GenBank [GenBank: JN133302 and JN133303].

### Experimental design

Due to the high seroprevalence of PCV1 in Flemish sows [6], viral replication and pathology cannot be studied by (oro)nasal inoculation of sows during gestation or by intrauterine inoculation of sows at insemination. Therefore, experimental PCV1 infections in foetuses have to be performed by direct in utero inoculation. Three conventional PCV1 seropositive Landrace sows were submitted to laparatomy at 55 days of gestation. Laparotomy of the sows was performed under anaesthesia as described previously [20]. In each of the three sows, three foetuses were inoculated: one foetus with the PCV1 cell culture strain CCL33; one with the PCV1 field isolate 3384 and one foetus with cell culture medium. The position in the uterus of the PCV1- and mock-inoculated foetuses, and their adjacent foetuses, is shown in Table 1. The inoculations were performed as described previously [20]. Briefly, the foetuses were inoculated by trans-uterine injection with 200 μL, containing 10^4.3 ^TCID_50 _of PCV1, into the peritoneal (100 μL) and amniotic (100 μL) cavities. For mock-inoculated foetuses, PK-15 cell culture medium (200 μL) was inoculated by trans-uterine injection with 200 μL into the peritoneal (100 μL) and amniotic (100 μL) cavities. The inoculated foetuses were marked with a synthetic, non-absorbable, superficial suture (Prolene^® ^2-0, Ethicon, Inc., Somerville, New Jersey, U.S.A.) on the exterior uterine wall. Antibiotics were administered to the sows before closure of the operation wound (Duphapen^® ^Strep, (Fort Dodge Animal Health Benelux, Netherlands), 10 mL intraperitoneally and 10 mL in the operation wound).

**Table 1:** PCV1- and mock-inoculated and their adjacent foetuses and results of PCR.

**Sow no**.	Foetus no.^a^	Inoculated with	PCR result^f^
S1	L5	NI^b^	-
	L6^c^	Mock	-
	R1	CCL33	+
	R2	NI^b^	-
	R3	NI^b^	-
	R4	3384	+
	R5^c^	NI^b^	-

S2	L1	Mock	-
	L2^d^	NI^b^	-
	R1	NI^b^	-
	R2	CCL33	+
	R3	3384	+
	R4^d^	NI^b^	-

S3	L1^e^	Mock	-
	R1	CCL33	+
	R2	3384	+
	R3^e^	NI^b^	-

^a ^Foetuses were identified by their position in the uterus. L = left horn; R = right horn.

Numbering is in sequence from ovary to cervix.

^b ^NI = not inoculated.

^c ^L6 and R5 were adjacent to each other.

^d ^L2 and R4 were adjacent to each other.

^e ^L1 and R3 were adjacent to each other.

^f ^PCR amplification of the *Rep *gene.

The sows were housed individually in A2 experimental units. The sows were observed daily for clinical signs and their rectal temperature was monitored daily during the first week after surgery. Twenty-one days post inoculation (dpi), the sows were humanly euthanized with an overdose of pentobarbitalum natricum [Natriumpentobarbital 20%^® ^40 mg/kg iv in the V. jugularis externa] (Kela Laboratoria, Hoogstraten, Belgium). Hysterectomy was performed and all foetuses were collected. The specific length of the tail ends of the sutures was used to determine the PCV1 strain the foetus was inoculated with.

All inoculated and non-inoculated foetuses were examined for gross lesions and tissue samples were collected from the heart, lungs, spleen, liver, kidneys, thymus, tonsils, ileum and cerebrum for histopathological examinations (haematoxylin and eosin staining), for virus titrations and for staining of infected cells by indirect immunofluorescence. Serum and abdominal fluid were collected as well. The serum samples of the sows were collected prior to surgery (pre-serum) and at the time of euthanasia (post-serum).

The animal experiments described in this study were authorized and supervised by the Ethical and Animal Welfare Committee of the Faculty of Veterinary Medicine of Ghent University.

### PCV1 isolation and titrations

Ten% (wt/vol) tissue suspensions (spleen, thymus, tonsils, ileum) and 20% (wt/vol) tissue suspensions (heart, lungs, liver, kidneys and cerebrum) were prepared in phosphate-buffered saline (PBS). For the PCV1-inoculated foetuses, the PCV1 titres in the above mentioned organs were determined by virus titration in PK-15 cells, as described before for PCV2 titration [20,21]. Briefly, PCV1-infected PK-15 cells were revealed by immunoperoxidase staining with using an optimal dilution of mono-specific anti-PCV1 swine polyclonal serum (produced in our laboratory) and peroxidase-labelled goat-anti-swine IgG (Jackson ImmunoReasearch, UK) as primary and secondary antibodies, respectively. For the mock-inoculated and non-inoculated foetuses, PCV1 titres were determined in the heart, lungs and spleen. The titration experiments were repeated independently for 3 times. For 10% suspensions, the detection limit of this technique was 10^2.0 ^TCID_50_/g tissue and for 20% suspensions, the detection limit was 10^1.7 ^TCID_50_/g tissue.

### Single immunofluorescence staining

The number of PCV1-positive cells in all of the collected organs of PCV1-inoculated foetuses and in the lungs of mock-inoculated and non-inoculated foetuses was determined by an indirect immunofluorescence staining (IIF), adapted from the technique described by Sanchez *et al*. [21]. Methanol-fixed cryostat sections were incubated with an optimal dilution of biotin-conjugated porcine anti-PCV1 polyclonal antibodies (pAbs). Subsequently, a 1:200 dilution of fluorescein isothiocyanate (FITC)-labelled streptavidin (Molecular Probes, Eugene, Oregon, USA) in PBS was applied. Both incubations were performed for 1 h at 37°C and sections were washed three times with PBS between the incubations. Finally, sections were incubated with Hoechst (Molecular Probes, Eugene, Oregon, USA) for 10 min followed by three washings with PBS. The specificity of the staining was confirmed by the deletion of primary antibody (anti-PCV1 pAb) and by the complete absence of fluorescence in the tissue sections of non-inoculated, age-matched foetuses. Stained tissue sections were mounted with a glycerol solution containing 1,4-diazobicyclo-2.2.2-octane (DABCO) anti-fading agent (Janssen Chimica, Beerse, Belgium). The number of PCV1-positive cells was determined in an area of 10 mm^2 ^of tissue by a LEICA DM/RBE fluorescence microscope (Leica Microsystems GmbH, Heidelberg, Germany) as described by Sanchez *et al*. [21]. Representative digital images of the stained tissue sections were made using the Olympus IX81 microscope connected with a Cell-M Live-Cell imaging module.

### Double immunofluorescence staining

To our knowledge, the target cells of PCV1 have not been characterized with cell markers yet. A cytokeratin marker monoclonal antibody (mAb) AE1/AE3 (Neomarkers, Fremont, CA) was used to identify epithelial cells [22]. Polyclonal rabbit antibodies against the human Von Willebrand Factor (pAb anti-human VWF) (DakoCytomation) were used to detect endothelial cells. Since PCV1 has previously been associated with cells showing macrophage morphology in pigs [9], the macrophage-marker mAb 41D3, detecting porcine sialoadhesin, was used [23,24]. Since mAb 41D3 is specific for macrophages, a mAb 74.22.15 (directed against SWC3) was used which detects not only macrophages but also monocytes, dendritic cells and granulocytes [25,26]. MAb 28.4.1 directed against IgM [27] was used as marker for B-lymphocytes. MAbs BB23-8E6 [28], 74.12.4 [29,30] and 76.2.11 [30-32] directed against CD3, CD4 and CD8, respectively, were used as markers for T-lymphocytes.

A double immunofluorescence staining for epithelial cells/endothelial cells/macrophages/B-lymphocytes/T-lymphocytes and PCV1 was performed in the lungs of PCV1-inoculated foetuses, adapted from the technique described by Sanchez *et al*. [26,33]. Briefly, methanol-fixed cryostat sections were first incubated with mAb AE1/AE3 (1:100), pAb VWF (1:25), mAb 41D3 (1:5), mAb anti-SWC3 (1:10), mAb anti-IgM (1:50), mAb anti-CD3 (1:50), mAb anti-CD4 (1:50) or mAb anti-CD8 (1:50) and then with FITC-labelled goat-anti-mouse IgG (1:200) (Molecular Probes, Eugene, Oregon, USA) or FITC-labelled goat-anti-rabbit IgG (1:200) (Molecular Probes). Afterwards, the sections were stained for PCV1 antigens by incubation with an optimal dilution of biotin-conjugated porcine anti-PCV1 pAbs. Subsequently, a 1:200 dilution of Texas Red-labelled streptavidin (Molecular Probes) in PBS was added. All antibodies were diluted in PBS and all incubations were performed for 1 h at 37°C. The sections were washed three times with PBS after each incubation with primary and secondary antibodies. Specificity of the staining for different cell markers (except for the endothelial marker) was demonstrated using an irrelevant, isotype matched mAb 1C11, 3H12 and 13D12 [34] and by the deletion of primary antibodies (pAb anti-human VWF and anti-PCV1 pAb), and by the complete absence of PCV1-specific fluorescence in tissue sections of non-inoculated, age-matched foetuses. The stained tissue sections were mounted as described above and PCV1-positive cells and double positive cells (cell marker and PCV1-positive cells) were quantitated as described previously [26,33] by using a LEICA DM/RBE fluorescence microscope (Leica Microsystems GmbH, Heidelberg, Germany). Representative digital images of stained preparations were made using the Olympus IX81 microscope connected with a Cell-M Live-Cell imaging module.

### Amplification of the PCV1 rep gene and sequencing

DNA was extracted from the heart and lung tissues of PCV1-inoculated, mock-inoculated and their adjacent foetuses by using a NucleospinR tissue kit (Macherey-Nagel). A set of primers (PF2: 5'-TTGCTGAGCCTAGCGACACC-3'; PR2: 5'-TCCACTGCTTCAAATCGGCC-3') was used to amplify a PCV1 349 bp replicase gene (Rep) fragment following the same methods as described by Larochelle *et al*. [35]. PCR products (Rep) were treated with Exonuclease I and Antarctic Phosphatase (New England Biolabs, Ipswich, USA) and used directly for cycle sequencing with a Big Dye Terminator Cycle sequencing kit V1.1 (Applied Biosystems, Foster City, USA). The cycle sequencing reaction products were purified using ethanol precipitation and separated on an ABI Genetic Analyzer 310 (Applied Biosystems, Foster City, USA). Sequence alignments were performed using bl2seq at http://blast.ncbi.nlm.nih.gov. To confirm the absence of porcine circovirus type 2 (PCV2) in PCV1-inoculated foetuses, amplification of PCV2 *capsid *was performed with the heart and lung tissues as described previously in Saha *et al*. [20].

### Serology

The PCV1-specific antibody titres in serum were determined by an immuno-peroxidase monolayer assay (IPMA) as described previously [6]. The PCV1 cell culture strain CCL33 was used as antigen. The foetuses were also checked for PCV2-specific antibodies by IPMA as described above but with PCV2 strain 1121 [36] as antigen. These assays were independently repeated 3 times.

The sow antibody titres against PCV1, PCV2 and porcine reproductive and respiratory syndrome virus (PRRSV) were determined by an IPMA (as described above) and the sow antibody titres against porcine parvovirus (PPV) were determined by a haemagglutination inhibition (HI) test as described elsewhere [37,38].

## Results

### Evaluation of the sows

The three sows were clinically healthy during the whole study period. No rise of rectal temperature was noticed in any of the three sows. The operation wounds were slightly swollen until 48 hours after the operations and somewhat painful at palpation. The PCV1- and PCV2-specific IPMA Ab titres of the three sows ranged from 40 to 640 and from 20,480 to 163,840, respectively in pre-serum and were identical in post-serum. The PRRSV-specific IPMA Ab titres and PPV-specific HI Ab titres in the pre-serum ranged from < 10 to 640 and from < 8 to 512, respectively. Seroconversion against PRRSV and PPV was not observed in any of the three sows.

### Gross examinations

**Figure 1:**
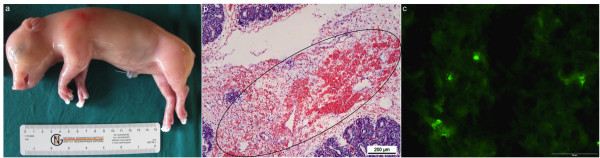
**Different aspects of PCV1-CCL33 replication after inoculation of a 55-day old foetus**. a) CCL33-inoculated foetus (S1R1) with a normal external appearance. b) Haematoxylin and eosin staining of the lungs of a CCL33-inoculated foetus (S1R1). Haemorrhages (indicated by a circle) in interlobular regions (magnification 10×). Bar = 200 μm. c) PCV1-positive cells in the lungs of CCL33-inoculated foetus (S1R1). Bar = 100 μm.

All PCV1-inoculated foetuses were normal in appearance and no evidence of gross pathological lesions was observed in any of the PCV1-inoculated foetuses (Figure 1a). The mock-inoculated and non-inoculated foetuses were also normal in appearance and no gross pathology was observed.

### Microscopic examinations

A haematoxylin and eosin (HE) staining was performed for different organs (heart, lungs, spleen, liver, kidney, thymus, cerebrum) of PCV1-inoculated and mock-inoculated foetuses. Microscopic lesions including severe haemorrhages in the interlobular regions were observed in the lung tissue of two foetuses (S1R1 and S2R2) inoculated with the cell culture PCV1 strain, CCL33 (Figure 1b). Microscopic lesions were not present in the other organs of these two foetuses. Microscopic lesions could not be observed in the third CCL33-inoculated foetus, the three 3384-inoculated foetuses and the mock-inoculated foetuses.

### PCV1 isolation and titrations

High PCV1 titres were found in the lungs of the foetuses S1R1 (10^4.7 ^TCID_50_/g tissue), S2R2 (10^4.6 ^TCID_50_/g tissue) and S3R1 (10^2.9 ^TCID_50_/g tissue) inoculated with the cell culture strain CCL33. All other organs of CCL33-inoculated foetuses were negative (< 10^1.7 ^TCID_50_/g tissue) by virus titration. All collected organs from the 3384-inoculated foetuses were negative (< 10^1.7 ^TCID_50_/g tissue) by virus titration. Mock-inoculated and non-inoculated foetuses were negative for PCV1.

### Single immunofluorescence staining

The number of PCV1-positive cells in the lungs of the three CCL33-inoculated foetuses, *i.e*. S1R1, S2R2 and S3R1, were 121, 31 and 28 cells/10 mm^2^, respectively (Figure 1c.). A low number of PCV1-positive cells/10 mm^2 ^was observed in the lungs of the three foetuses inoculated with the field strain 3384 (4 (S1R4), 13 (S2R3) and 1 (S3R2) cells/10 mm^2 ^respectively). PCV1-positive cells were also observed in several other organs such as spleen, liver (S1R1); spleen, liver and tonsils (S2R2); and heart and thymus (S2R3) and the numbers varied between 1 and 6 cells/10 mm^2 ^of tissues (Table 2). PCV1-positive cells were not observed in the lungs of the mock-inoculated and non-inoculated foetuses.

**Table 2:** Quantification of PCV1 positive cells in different foetal organs collected at 21 days post-PCV1 inoculation.

Strain	**Sow no**.	Inoculated foetus^a^	Number of PCV1 positive cells/10 mm^2 ^tissue								
			
			Heart	Lungs	Spleen	Liver	Kidney	Thymus	Tonsils	Ileum	Cerebrum
CCL33	S1	R1	-	121	6	4	-	-	-	-	-
CCL33	S2	R2	-	31	3	2	-	-	1	-	-
CCL33	S3	R1	-	28	-	NA	NA	NA	-	-	NA
3384	S1	R4	-	4	-	-	-	-	NA	NA	-
3384	S2	R3	4	13	-	-	-	3	-	-	-
3384	S3	R2	-	1	-	-	-	-	-	NA	-

^a ^Foetuses were identified by their position in the uterus. R = right horn. Numbering is in sequence from ovary to cervix.

NA: not available; -: no positive cells detected

### Double immunofluorescence staining (DIF)

**Figure 2:**
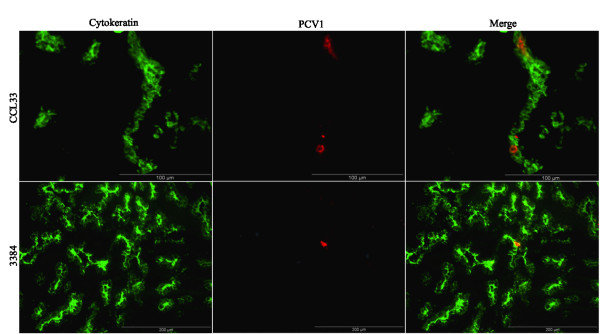
**Co-localization of PCV1 in the epithelial cells**. Co-localization of PCV1 in the epithelial cells of the lungs of a CCL33-inoculated foetus (Bar = 100 μm) and a 3384-inoculated foetus (Bar = 200 μm) collected at 21 days post inoculation (76 days of gestation). Methanol-fixed cryostat sections were incubated (as described in Materials and Methods) with a cytokeratin marker monoclonal antibody AE1/AE3 to stain epithelial cells (green fluorescence). Cells containing PCV1 antigens were localized using biotin-labelled mono-specific porcine anti-PCV1 polyclonal antibodies (red fluorescence).

**Figure 3:**
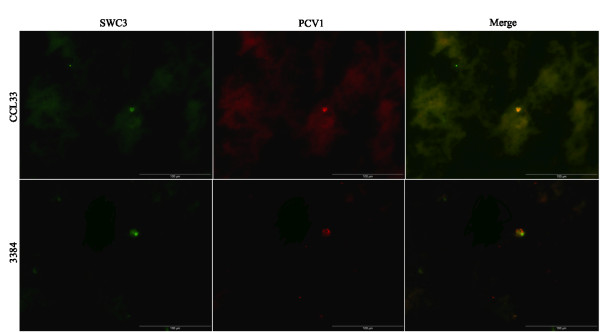
**Co-localization of PCV1 in monocytes (SWC3+ cells)**. Co-localization of PCV1 in monocytes (SWC3+ cells) of the lungs of a CCL33-inoculated foetus (Bar = 100 μm) and a 3384-inoculated foetus (Bar = 100 μm) collected at 21 days post inoculation (76 days of gestation). Methanol-fixed cryostat sections were incubated (as described in Materials and Methods) with a mAb anti-SWC3 (74.22.15) (green fluorescence). Cells containing PCV1 antigens were localized using biotin-labelled mono-specific porcine anti-PCV1 polyclonal antibodies (red fluorescence).

The PCV1 antigens were mainly (97%) localized in the epithelial cells of the lungs (Figure 2) of the PCV1-inoculated foetuses. The other 3% of the PCV1 antigens were localized in SWC3^+ ^cells (cells of the monocytic lineage) of the lungs (Figure 3). No co-localization of PCV1 antigens was observed in endothelial cells, 41D3^+ ^macrophages, B-lymphocytes and T-lymphocytes (data not shown).

### Amplification of PCV1 Rep gene and sequencing

In order to confirm that the foetuses had been inoculated with the specific PCV1 strain and were not contaminated with PCV1 from neighbouring foetuses (as mentioned in Table 1), a PCV1 Rep fragment was amplified using heart and/or lung tissue from the infected foetuses (21 days after inoculation) followed by sequencing. PCR and DNA sequencing recovered CCL33 and 3384 sequences from CCL33- and 3384-inoculated foetuses, respectively. No evidence for mixed samples (e.g. containing more than one PCV1 strain) was found. All the adjacent foetuses of PCV1-inoculated foetuses were negative in PCR assays. All the mock-inoculated foetuses and their adjacent foetuses were also negative in PCR assays (Table 1). All the PCV1-inoculated foetuses were also negative for PCV2 in PCR assays.

### Serology

All the PCV1-inoculated foetuses had a very low anti-PCV1 IPMA Ab titre of 10 to 40, except one foetus (S2R2) inoculated with CCL33, which had a titre of 160. The mock-inoculated and non-inoculated foetuses were negative (< 10) for PCV1-specific IPMA Ab. All the PCV1-inoculated and mock-inoculated foetuses were negative (< 10) for PCV2-specific IPMA Ab.

## Discussion

In this study, the virological and pathological outcomes were examined in immuno-incompetent porcine foetuses after inoculation with PCV1 at 55 days of gestation.

The PCV1 cell culture strain CCL33 was found to be pathogenic to porcine foetuses inoculated at 55-days of foetal life. Severe haemorrhages were present in the lungs of two out of three CCL33 inoculated foetuses. These lesions correlated well with the highest PCV1 titres (10^4.7 ^TCID_50_/g and 10^4.6 ^TCID_50_/g) in the lungs. The lungs with the lower level of virus replication (10^2.9 ^TCID_50_/g) did not have histopathological changes. Haemorrhages in the lungs can be explained by the fact that due to the high PCV1 replication in the epithelial cells, there might be the release of inflammatory mediators, which may ultimately lead to the increased permeability of the blood vessels followed by leakage of blood or haemorrhage. Under the conditions of the present study, the PCV1 field strain 3384 was non-pathogenic to porcine foetuses. This suggests that a high PCV1 load could be essential to induce pathology in porcine foetuses. Several experimental studies had been performed in the past to study the pathogenesis of PCV1 infections in 1-day old, 2-days-old, 1 month old and 9 months old pigs [8,9,14] but these experimental studies failed to reproduce disease in pigs. However, under the conditions of the present study, it was demonstrated that PCV1 is pathogenic for porcine foetuses. More research is needed to determine the pathogenicity of PCV1.

Previously, Allan *et al*. [9] showed the distribution of PCV1 antigens by virus isolation and IIF staining in different organs of pigs, to be predominantly in the lungs, although the level of PCV1 replication was not quantitatively determined in that study. Our study showed that the lung tissue was the main target organ of replication of CCL33 strain, as this PCV1 strain could only be isolated with high titres (up to 10^4.7 ^TCID_50_/g) from the lungs. The field strain 3384 could not be isolated from any of the inoculated foetuses, although they were clearly PCR positive. These findings show that the replication kinetics of the strains CCL33 and 3384 are different from each other. Sequence comparison of the Rep and capsid of CCL33 and 3384 revealed one synonymous nucleotide substitution in the Rep and 4 amino acid (aa) differences (at aa positions 69, 72, 74 and 116) in the capsid protein (data not shown), suggesting that these aa differences in the capsid protein could be responsible for the different replication kinetics of these two strains in porcine foetuses. Further research may clarify this issue.

Single immunofluorescence staining revealed that moderate (up to 121 cells/10 mm^2 ^of tissues) and low numbers (up to 13 cells/10 mm^2^) of PCV1 positive cells were present in the lungs of the CCL33-inoculated and the 3384-inoculated foetuses, respectively, although no PCV1 could be isolated from the lungs of the 3384-inoculated foetuses. Moreover, all the other organs of the PCV1-inoculated foetuses were negative in virus isolation, whereas PCV1 antigens were found at a low level (up to 6 cells/10 mm^2^) in the heart, liver, spleen, thymus and tonsils. These results suggest that immunostaining of PCV1 is a more sensitive technique than virus isolation and titration for the detection of PCV1. Comparably, a previous study of McNeilly *et al*. [39] with PCV2 indicated that immunostaining of PCV2 is a more sensitive technique than virus isolation for the detection of PCV2 in porcine tissues.

The double staining of PCV1 and different cell markers established that the PCV1 antigens were mainly localized in the lung epithelial cells and not in endothelial cells, macrophages or lymphocytes of the lungs of PCV1-inoculated foetuses. This is in contradiction with previous observations in new-born piglets [9,16], where it was shown that PCV1 is mainly present in non-epithelial cells, morphologically resembling macrophages. It could be possible that the target cells for PCV1 in foetal life might be different from the target cells for PCV1 in newborn pigs, as previously shown for PCV2 by Sanchez *et al*. [26]. PCV1 needs cellular DNA polymerases of actively dividing cells to replicate [40] and presumably epithelial cell types possess more mitotic activity in immuno-incompetent porcine foetuses than in newborn pigs. The remaining 3% of the PCV1 antigens were localized in SWC3^+ ^cells, which were 41D3^-^. It could be possible that due to high PCV1 replication in the lungs, there were newly infiltrating monocytes (SWC3^+^, 41D3^-^). This study also indicates that PCV1 and PCV2 have different cell tropism during foetal life, since PCV2 replicates mainly in the cardiomyocytes and macrophages of the heart tissue [26] and PCV1 targets mainly the epithelial cells of the lungs.

In previous studies with PCV2 [20,21,41,42], it was shown that PCV2 does not spread rapidly from one foetus to another. Under the conditions of the present study, no intrauterine spread of PCV1 from PCV1-inoculated to non-inoculated foetuses was observed. Immuno-competency in porcine foetuses develops at around 80 days of gestation [43]. More specifically, porcine foetuses are able to mount a protective immune response against small, non-enveloped, single-stranded DNA viruses like PCV1, PCV2 or PPV when they are infected after 70 days of gestation [20,21,44]. Foetuses were inoculated with PCV1 strains at 55 days of foetal life and their immune response to PCV1 was confirmed by determining the PCV1-specific IPMA Ab titre. All PCV1-inoculated foetuses developed very low anti-PCV1 antibody titres (10 to 40), except one foetus, which had a titre of 160 that was inoculated with CCL33 strain.

## Conclusions

From this study, it can be concluded that PCV1 can replicate and may produce pathology in the lungs of porcine foetuses inoculated at 55-days of foetal life. More research is needed to confirm the pathogenic character of PCV1 for porcine foetuses.

## Authors' contributions

DS: contributed during laparotomy and study design, collected and processed the samples, evaluated the data, wrote the manuscript. DL: helped during laparotomy, collected the samples, helped with the writing of the manuscript. RD: helped with the histopathological examinations. JD: contributed to the PCR and sequencing, helped with the writing of the manuscript. HJ: obtained the funding, contributed to the study design, laparotomy, evaluated the data, critically revised the manuscript. All authors read and approved the final manuscript.
